# Comparison of Optic Nerve Head Parameters in Alcoholic Liver Disease Patients With Age-Matched Controls

**DOI:** 10.7759/cureus.104366

**Published:** 2026-02-27

**Authors:** Sujata Priyambada, Bhabani Shankar Sahu, Divya Mohindru, Chandan Dixit, Srijit Mohanty

**Affiliations:** 1 Ophthalmology, Hi-Tech Medical College and Hospital, Rourkela, IND; 2 Internal Medicine, Hi-Tech Medical College and Hospital, Rourkela, IND

**Keywords:** alcoholic liver disease, alcohol use disorder, optical coherence tomography, optic nerve head, retinal nerve fiber layer

## Abstract

Alcoholic liver disease (ALD) is associated with systemic neurodegeneration mediated by oxidative stress, mitochondrial dysfunction, and chronic inflammation. As the retina and optic nerve head represent extensions of the central nervous system, they may provide a non-invasive window for detecting alcohol-related neuroretinal damage. This retrospective case-control study compared optic nerve head and peripapillary retinal nerve fiber layer (pRNFL) parameters between 100 patients with ALD and 100 age- and sex-matched healthy controls who underwent comprehensive ophthalmic evaluation and spectral-domain optical coherence tomography imaging. Average and quadrant-wise pRNFL thicknesses were analyzed across predefined age groups using independent samples t-tests. Patients with ALD demonstrated a significantly reduced average pRNFL thickness compared with controls (p < 0.05), with more pronounced thinning observed in younger adults aged 21-40 years. Quadrant-wise analysis revealed predominant involvement of the nasal and temporal quadrants, with relative sparing of the inferior quadrant. An age-related decline in pRNFL thickness was observed in both groups; however, the rate of decline was greater among patients with ALD. These findings suggest that ALD is associated with early and progressive neuroretinal degeneration, distinct from the typical pattern seen in glaucomatous optic neuropathy, and highlight the potential role of spectral-domain optical coherence tomography as a non-invasive tool for detecting subclinical neuroretinal damage in patients with ALD.

## Introduction

Alcoholic liver disease (ALD) encompasses a spectrum of hepatic disorders ranging from steatohepatitis to cirrhosis and hepatocellular carcinoma and is emerging as a leading cause of chronic liver disease worldwide, reflecting evolving societal and cultural patterns of alcohol consumption [1]. Changes in alcohol use patterns include earlier age of initiation, increased consumption, binge drinking, and shifting social norms [2]. Globally, alcohol use disorder (AUD) accounts for approximately 3.3 million deaths annually and represents one of the leading causes of preventable mortality in Europe and the United States [3]. In India, alcohol contributes to nearly 43% of cirrhosis cases, with 10%-30% progressing to hepatocellular carcinoma, underscoring its significant public health burden [4].

The systemic effects of chronic alcohol consumption extend beyond hepatic injury. Alcohol-induced oxidative stress, mitochondrial dysfunction, and disruption of the blood-brain barrier contribute to neuronal inflammation and glial injury. Alcoholic steatohepatitis further promotes systemic inflammation through the release of pro-inflammatory cytokines and toxic lipid metabolites that may traverse the blood-brain barrier and accelerate neurodegeneration [5-9]. These mechanisms highlight the existence of a hepatic-neural axis, wherein hepatic dysfunction may influence central nervous system integrity.

Several studies have explored the association between chronic alcohol consumption and ocular parameters. Alcohol intake has been linked to elevated intraocular pressure (IOP), open-angle glaucoma (OAG), and ocular hypertension in some populations [10-13]. However, large-scale studies, including the Beaver Dam Eye Study, have reported inconsistent associations between alcohol consumption and glaucoma risk [14,15]. Notably, findings from the Gutenberg Health Study demonstrated that higher alcohol consumption was associated with thinning of the peripapillary retinal nerve fiber layer (pRNFL), potentially reflecting alcohol-induced mitochondrial damage and retinal neurodegeneration [16].

The retina and optic nerve head (ONH) are considered extensions of the central nervous system due to their embryological origin and microvascular architecture [17]. Thinning of the retinal nerve fiber layer (RNFL) and degeneration of retinal ganglion cells have been documented in various neurodegenerative and psychiatric disorders, suggesting that retinal imaging may serve as a surrogate marker of subclinical neural injury [18,19]. Spectral-domain optical coherence tomography (SD-OCT) offers a non-invasive and reproducible method for quantifying structural retinal changes.

In this retrospective case-control study, we aimed to quantitatively compare average and quadrant-wise pRNFL thickness and ONH parameters measured by SD-OCT between patients with ALD and age-matched healthy controls. We hypothesized that patients with ALD would demonstrate significantly reduced average and sectoral pRNFL thickness and altered ONH parameters compared to controls. Age-stratified and quadrant-wise analyses were predefined to evaluate regional patterns of RNFL thinning.

## Materials and methods

Study design and setting

This retrospective, cross-sectional, case-control study was conducted in the Department of Ophthalmology at Hi-Tech Medical College and Hospital, Rourkela. Medical and imaging records of eligible participants evaluated between January 2024 and June 2025 were reviewed. The study aimed to compare average and quadrant-wise pRNFL thickness and ONH parameters between patients with ALD and age- and sex-matched healthy controls.

Ethical approval

Ethical approval was obtained from the Institutional Ethics Committee of Hi-Tech Medical College and Hospital, Rourkela (Approval No. HMCHR/ETHICS/2025/28 dated August 8, 2025). The committee approved the retrospective analysis of existing patient records. As this was a record-based study, no prospective recruitment or additional investigations were performed, and patient confidentiality was maintained in accordance with institutional and ethical guidelines.

Participant selection

A non-probability consecutive sampling technique was employed. All eligible patients diagnosed with ALD during the study period who met the inclusion criteria were included as cases. Age- and sex-matched healthy controls were selected consecutively from hospital attendees during the same period using frequency matching rather than individual (pairwise) matching.

Patients aged 21-80 years diagnosed with ALD based on the Child-Pugh cirrhosis criteria (score > 5), with alcohol as the sole etiology of liver disease, were included. The severity of liver disease was categorized according to the Child-Pugh classification as documented in medical records. However, subgroup analysis based on the Child-Pugh class was not performed due to limited distribution across severity categories.

The duration of alcohol consumption was recorded from available medical documentation. However, quantitative lifetime alcohol exposure (e.g., grams per day or cumulative units) was not uniformly available due to the retrospective nature of the study. Controls were healthy individuals with best-corrected visual acuity (BCVA) of 6/9 or better, no history of alcohol or substance abuse, and no known systemic or ocular disease.

Participants were excluded if they had glaucoma, ocular hypertension, optic neuropathy, maculopathy, significant refractive error greater than ±4.00 diopters, ocular inflammation, ocular trauma, and prior intraocular surgery, or were receiving medications known to affect the optic nerve (e.g., ethambutol). Individuals with non-ALD, diabetes mellitus, hypertension, neurodegenerative disorders, or any retinal or optic nerve pathology detected on clinical examination were also excluded to minimize confounding factors known to influence pRNFL thickness. Smoking status and nutritional parameters were not consistently documented and, therefore, were not included in the analysis. Although major systemic and ocular confounders were excluded through predefined criteria, residual confounding from unmeasured variables such as cumulative alcohol exposure, smoking status, and nutritional deficiencies cannot be completely ruled out.

Ophthalmic evaluation

All participants underwent a comprehensive ophthalmic evaluation, including BCVA assessment, color vision testing, and contrast sensitivity measurement. IOP was measured using Goldmann applanation tonometry, and anterior chamber angle evaluation was performed using a Sussman goniolens. Slit-lamp biomicroscopy with a +90 diopter lens and indirect ophthalmoscopy were performed to assess the posterior segment. Standard automated perimetry was conducted to exclude visual field defects. These examinations ensured exclusion of pre-existing retinal or optic nerve pathology.

Optical coherence tomography imaging

SD-OCT was performed using the Topcon 3D OCT Maestro 2 platform. The disc scan protocol covered a 6.0 × 6.0 mm area using 512 × 256 super-pixels. Fixation was achieved using a central cross or an external fixation target. All scans were acquired by a trained ophthalmic technician using a standardized imaging protocol, and device calibration was maintained according to manufacturer guidelines throughout the study period.

Only scans with a signal strength index greater than 40 were included to ensure adequate image quality. Scans with motion artifacts, segmentation errors, or incomplete data were excluded. To avoid inter-eye correlation bias, only the right eye of each participant was included in the analysis.

The primary outcome measures were average pRNFL thickness (µm) and quadrant-wise pRNFL thickness (superior, inferior, nasal, and temporal). Secondary outcome measures included ONH parameters such as disc area, rim area, cup-to-disc ratio, and cup volume. Age-stratified and quadrant-wise analyses were predefined prior to statistical evaluation.

Statistical analysis

Age and sex were matched at the group level using frequency matching; therefore, comparisons were performed using independent samples t-tests. Data were entered into Microsoft Excel (Microsoft Corporation, Redmond, WA, USA; Version 2019) and analyzed using IBM SPSS Statistics Version 25 (IBM Corp., Armonk, NY, USA). Quantitative variables were expressed as mean ± standard deviation.

Normality of data distribution was assessed using the Shapiro-Wilk test. Homogeneity of variances was evaluated using Levene’s test before the application of parametric tests. Differences in average and quadrant-wise pRNFL thickness and ONH parameters between ALD cases and controls were analyzed using the independent samples t-test.

Age-stratified subgroup analysis was performed to assess variations in pRNFL thickness across predefined age categories. Given multiple quadrant-wise comparisons, findings were interpreted with consideration of potential type I error inflation. A p-value less than 0.05 was considered statistically significant.

Records with incomplete OCT scans or inadequate image quality were excluded from the final analysis. No imputation for missing data was performed.

Sample size calculation

The sample size was calculated using the formula for comparison of two independent means:

$n=\frac{2\left( Z_{\alpha/2}+Z_{\beta} \right)^{2}\sigma^{2}}{d^{2}}$

where *Z*_*α*/2_ was 1.96 (95% confidence level) and *Z_β_* was 0.84 (80% power). The estimated standard deviation (*σ*) of average pRNFL thickness was assumed to be 12 µm based on previously published OCT studies in similar populations, and the minimum clinically significant difference (d) between groups was considered to be 5 µm. Based on this calculation and record availability, 100 cases and 100 controls were included in the study.

## Results

Demographic profile

Of the 200 participants included in the study, 100 patients (50.0%) had ALD and 100 participants (50.0%) served as healthy controls. The mean age was 42.8 ± 7.6 years in the ALD group and 45.2 ± 8.1 years in the control group. Men constituted 75 patients (75.0%) in the ALD group and 67 participants (67.0%) in the control group. Rural residence was observed in 67 patients (67.0%) among cases and 53 participants (53.0%) among controls. Most participants were married, including 83 cases (83.0%) and 80 controls (80.0%). Detailed sociodemographic characteristics are presented in Table 1.

**Table 1:** Sociodemographic characteristics of study participants. *p-value calculated using the independent samples t-test. p-values for categorical variables calculated using the Chi-squared test. ^†^p-values not shown for education level and occupation as these variables were not used for matching and are presented for descriptive purposes only. SD: standard deviation

Variable	Category	Cases (n = 100)	Controls (n = 100)	p-value
Age (years)	Mean ± SD	42.8 ± 7.6	45.2 ± 8.1	0.032*
Sex	Male	75 (75.0%)	67 (67.0%)	0.275
	Female	25 (25.0%)	33 (33.0%)	
Residence	Urban	33 (33.0%)	47 (47.0%)	0.061
	Rural	67 (67.0%)	53 (53.0%)	
Education level	Illiterate	8 (8.0%)	5 (5.0%)	—^†^
	Primary	40 (40.0%)	20 (20.0%)	
	Secondary	34 (34.0%)	34 (34.0%)	
	Graduate & above	18 (18.0%)	41 (41.0%)	
Occupation	Unemployed	10 (10.0%)	13 (13.0%)	—^†^
	Manual labor	38 (38.0%)	28 (28.0%)	
	Skilled worker	30 (30.0%)	35 (35.0%)	
	Professional	22 (22.0%)	24 (24.0%)	
Marital status	Married	83 (83.0%)	80 (80.0%)	0.716
	Unmarried	17 (17.0%)	20 (20.0%)	

RNFL parameters across age groups

For all age groups, the average pRNFL thickness in ALD patients was lower than that of controls (Table 2). Statistically significant differences in average RNFL thickness were observed in the 21-30-year (p = 0.048), 31-40-year (p = 0.039), and 61-70-year (p = 0.0168) age groups.

**Table 2:** Comparison of RNFL parameters between cases and controls across age groups. Values are expressed as mean ± standard deviation (SD). Between-group comparisons were performed using the independent samples t-test. A p-value < 0.05 was considered statistically significant. RNFL: retinal nerve fiber layer

Age group	RNFL parameter	Cases (mean ± SD)	Controls (mean ± SD)	t(df)	p-value
21–30 yrs	Superior	148.61 ± 7.41	150.00 ± 12.58	t(df)	0.0357
	Inferior	151.83 ± 7.67	156.78 ± 4.21	t(df)	0.8442
	Nasal	99.65 ± 8.12	108.44 ± 7.76	t(df)	0.016
	Temporal	91.48 ± 10.39	97.89 ± 11.55	t(df)	0.049
	Average	124.48 ± 13.14	134.39 ± 7.96	t(df)	0.048
31–40 yrs	Superior	144.13 ± 10.76	148.86 ± 14.87	t(df)	0.1925
	Inferior	149.32 ± 9.21	152.38 ± 12.29	t(df)	0.0983
	Nasal	93.52 ± 13.08	96.52 ± 17.83	t(df)	0.0498
	Temporal	84.45 ± 10.11	87.14 ± 15.31	t(df)	0.0417
	Average	116.19 ± 9.60	124.05 ± 2.49	t(df)	0.0391
41–50 yrs	Superior	138.83 ± 12.03	140.71 ± 12.06	t(df)	0.0313
	Inferior	146.33 ± 9.22	149.29 ± 9.62	t(df)	0.1163
	Nasal	90.79 ± 7.25	95.75 ± 11.20	t(df)	0.5311
	Temporal	75.42 ± 7.09	78.71 ± 15.56	t(df)	0.1469
	Average	115.83 ± 6.51	118.54 ± 10.38	t(df)	0.1486
51–60 yrs	Superior	123.76 ± 6.50	129.32 ± 7.40	t(df)	0.7654
	Inferior	127.43 ± 5.11	131.68 ± 8.37	t(df)	0.8022
	Nasal	85.57 ± 8.85	89.43 ± 9.70	t(df)	0.0001
	Temporal	71.05 ± 8.33	74.43 ± 9.18	t(df)	0.1962
	Average	108.29 ± 6.37	106.32 ± 5.70	t(df)	0.289
61–70 yrs	Superior	123.50 ± 8.32	127.69 ± 6.52	t(df)	0.6483
	Inferior	124.50 ± 6.02	129.15 ± 7.34	t(df)	0.9350
	Nasal	81.25 ± 5.57	82.62 ± 5.03	t(df)	0.0732
	Temporal	69.63 ± 3.70	70.85 ± 4.76	t(df)	0.1041
	Average	104.88 ± 6.49	105.38 ± 4.87	t(df)	0.0168

In the 21-30-year age group, cases demonstrated significantly thinner RNFL in the superior, nasal, and temporal quadrants compared with controls (p < 0.05). The average RNFL thickness was 124.48 ± 13.14 µm in cases and 134.39 ± 7.96 µm in controls, suggesting early subclinical RNFL thinning in younger ALD patients. In the 31-40-year age group, RNFL thinning persisted, with statistically significant reductions noted in the nasal, temporal, and average RNFL parameters (p < 0.05). Although the 41-50-year age group showed lower mean RNFL values across all quadrants in cases compared with controls, these differences were not statistically significant (p > 0.05).

Age-stratified analysis demonstrated statistically significant thinning of average pRNFL thickness in younger age groups, particularly between 21 and 50 years. In the 51-60-year age group, average pRNFL thickness in cases was comparable to, and marginally higher than, controls, with no statistically significant difference observed. In older age groups, differences were smaller and inconsistent. A comparison of average and quadrant-wise RNFL parameters across different age groups between cases and controls is presented in Table 2. For the 61-70-year age group, a statistically significant difference was observed in average RNFL thickness between cases (104.88 ± 6.49 µm) and controls (105.38 ± 4.87 µm) (p = 0.0168), although individual quadrant-wise differences were not statistically significant.

Quadrant-wise pattern of RNFL thinning

**Figure 1:**
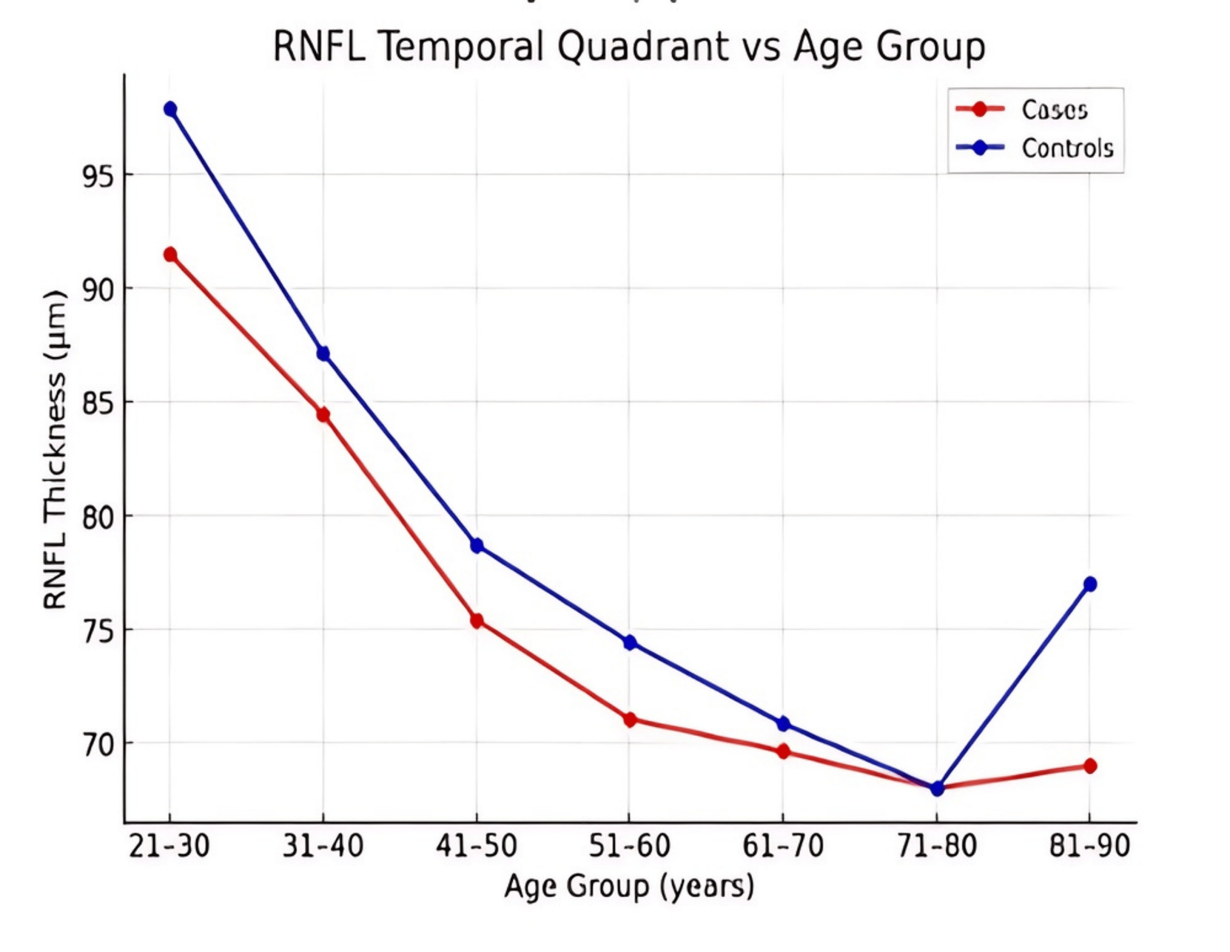
Line graphs showing the age-related decline in retinal nerve fiber layer (RNFL) thickness across the temporal quadrant. Data are presented as mean values across age groups. Statistical comparison between groups was performed using the independent samples t-test, with p < 0.05 considered statistically significant. All graphs are original data visualizations created by the authors for this study. pRNFL: peripapillary retinal nerve fiber layer

**Figure 2:**
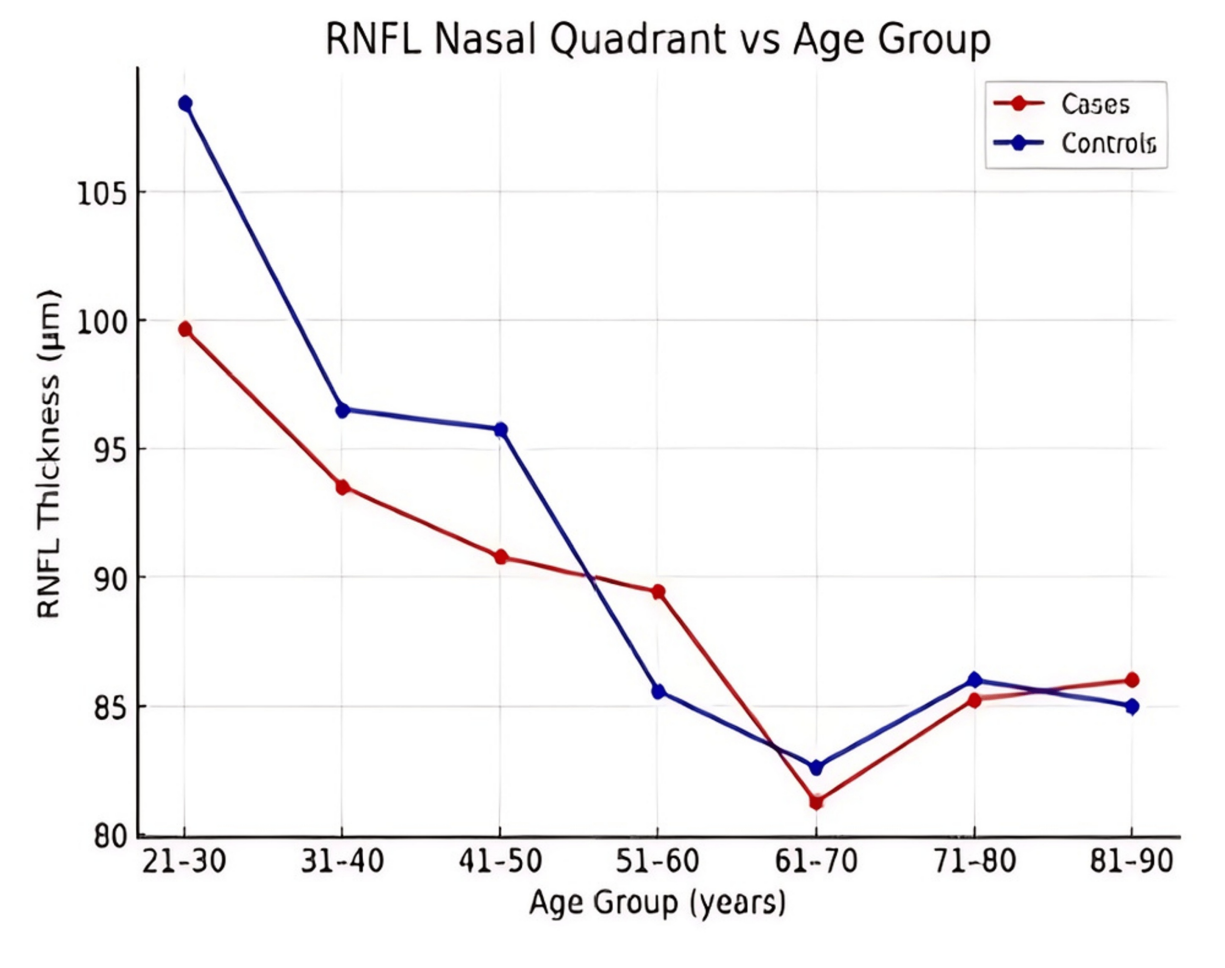
Line graph depicting age-related variation in retinal nerve fiber layer (RNFL) thickness in the nasal quadrant in patients with alcoholic liver disease (ALD) compared with controls.

**Figure 3:**
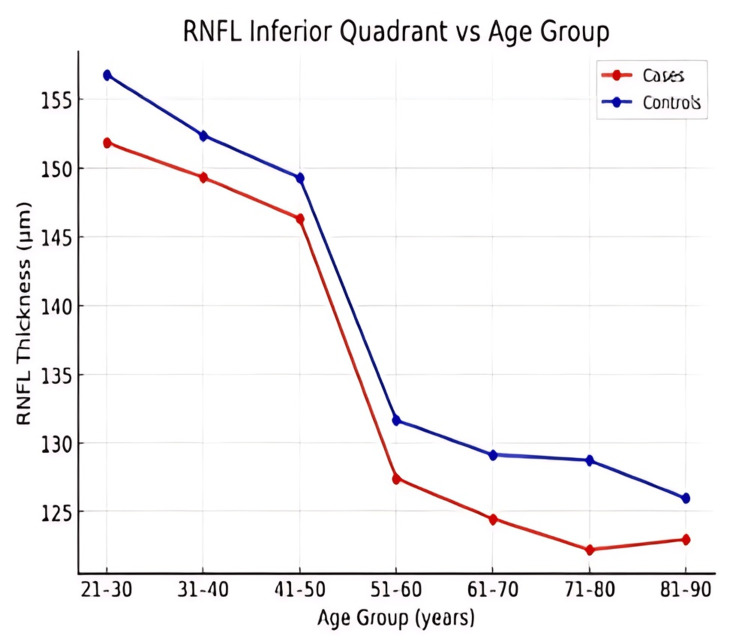
Line graph illustrating age-related trends in retinal nerve fiber layer (RNFL) thickness in the inferior quadrant in patients with alcoholic liver disease (ALD) and controls.

**Figure 4:**
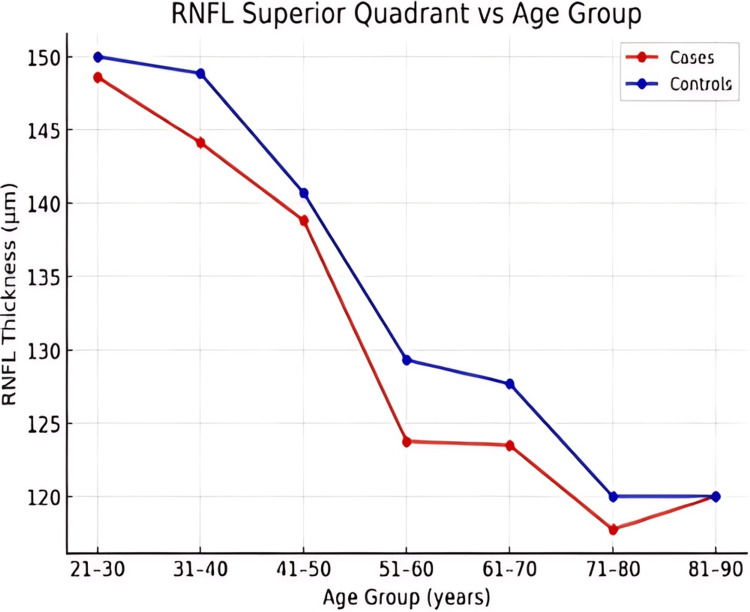
Line graph demonstrating age-related decline in retinal nerve fiber layer (RNFL) thickness in the superior quadrant in patients with alcoholic liver disease (ALD) and healthy controls.

Across all age groups, RNFL thickness was consistently lower in patients with ALD compared to controls in the superior, inferior, nasal, and temporal quadrants (Figures 1-4). However, the magnitude and pattern of thinning varied between quadrants.

In younger adults, the nasal and temporal quadrants demonstrated the most consistent and early RNFL thinning (Figures 1, 2). The superior quadrant showed a gradual decline in RNFL thickness with advancing age (Figure 4), whereas the inferior quadrant appeared relatively preserved across age groups (Figure 3).

Trend analysis

**Figure 5:**
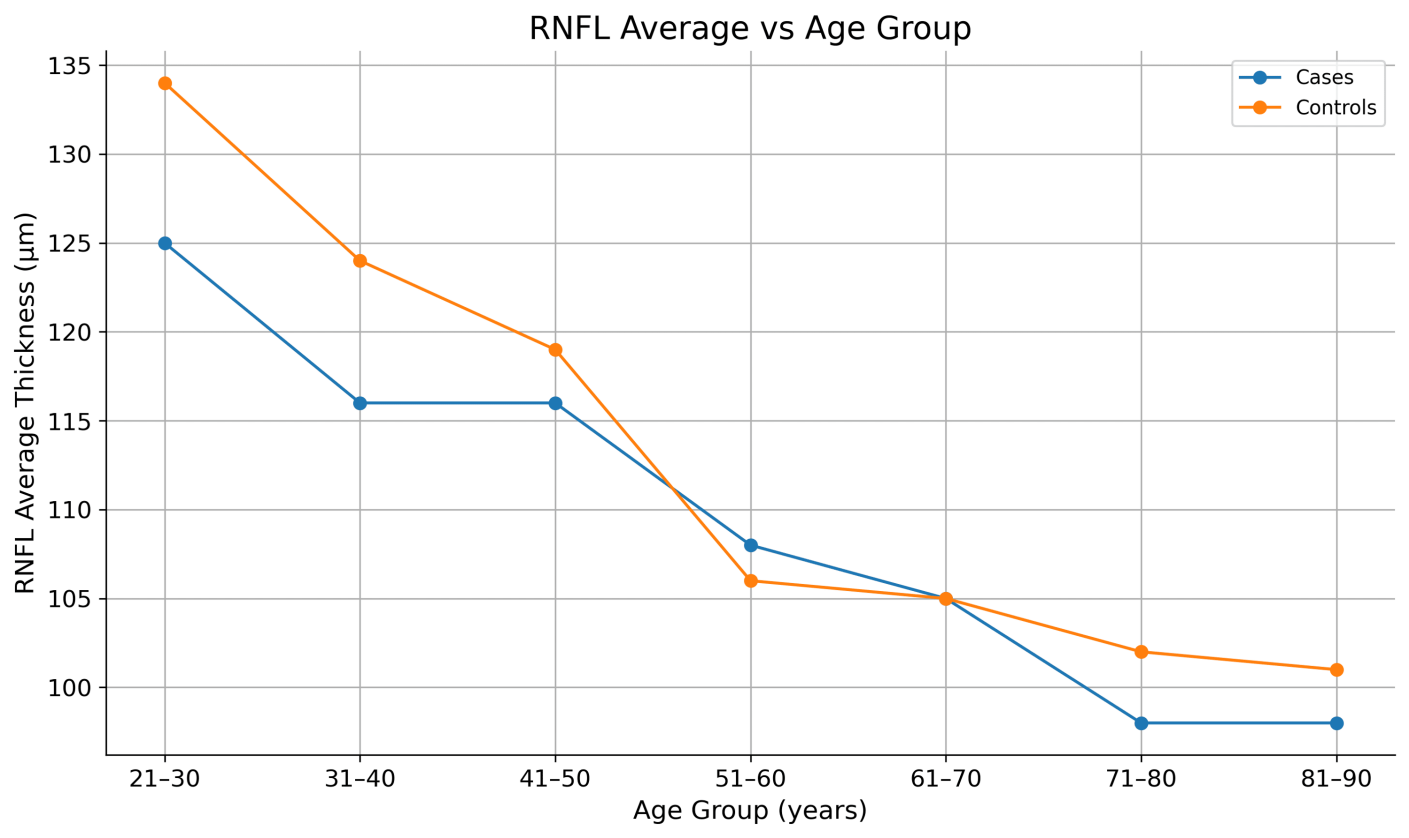
Line graph showing age-related changes in average retinal nerve fiber layer (RNFL) thickness in patients with alcoholic liver disease (ALD) compared with controls. The figure depicts the average pRNFL thickness across all quadrants. pRNFL: peripapillary retinal nerve fiber layer

Both cases and controls exhibited an age-related decline in RNFL thickness; however, the rate of decline was more pronounced in ALD patients (Figures 1-5). Younger ALD patients (21-40 years) demonstrated early RNFL loss, which progressively worsened with increasing age.

After 60 years of age, RNFL thickness values in cases and controls became comparable (Figure 5), suggesting that physiological age-related RNFL loss in controls approaches the pathological loss observed in ALD patients. The overall pattern of RNFL thinning was diffuse rather than quadrant-specific, resembling a neurodegenerative process rather than a glaucomatous pattern, with early involvement predominantly affecting the nasal and temporal fibers.

## Discussion

The present study demonstrated a significant reduction in average pRNFL thickness in patients with ALD compared with age- and sex-matched healthy controls, with the most pronounced thinning observed in younger adults aged 21-40 years. These findings indicate an association between ALD and reduced pRNFL thickness, suggesting possible early neuroretinal involvement. The greater difference observed in younger individuals is clinically notable and may reflect evolving alcohol consumption patterns, including earlier initiation and binge drinking behaviors [20].

The retina and ONH are recognized as structural extensions of the central nervous system due to their shared embryologic origin and microvascular characteristics [17,18]. Chronic alcohol exposure has been associated with oxidative stress, mitochondrial dysfunction, excitotoxicity, and neuroinflammation within the brain [6-9]. In the context of ALD, systemic inflammation and metabolic dysregulation may contribute to neuronal vulnerability. Although the present findings are consistent with these proposed mechanisms, direct causal pathways cannot be established in this retrospective analysis.

Previous studies have reported similar structural retinal alterations in individuals with AUD and liver disease. Akdemir et al. observed pRNFL thinning in patients with cirrhosis [21], while Álvarez-Sesmero et al. reported reduced RNFL thickness in individuals with AUD, even following abstinence [22]. Additional SD-OCT studies have demonstrated RNFL and macular thinning in alcohol-dependent populations [23-25]. The present study adds to the existing literature by incorporating age-stratified analysis, suggesting that retinal structural changes may be detectable earlier in life among patients with ALD.

The quadrant-wise pattern of pRNFL thinning observed in this cohort-predominantly involving the nasal and temporal quadrants with relative sparing of the inferior quadrant-was not uniformly significant across all age groups and showed attenuation in older participants. While this distribution differs from the classic superior-inferior pattern described in glaucomatous optic neuropathy [26], the interpretation should be made cautiously, as quadrant-wise differences were inconsistent and largely non-significant in older age strata. These findings may suggest a non-glaucomatous pattern of retinal involvement, rather than pressure-mediated optic nerve damage, but do not allow definitive differentiation from glaucomatous or other optic neuropathies. Similar non-glaucomatous patterns of RNFL thinning have been reported in other neurodegenerative and psychiatric conditions, supporting the potential role of retinal imaging as a surrogate marker of broader neural involvement [18,19].

An age-related decline in pRNFL thickness was observed in both cases and controls, consistent with established normative data [26]. However, the reduction appeared more pronounced in patients with ALD, suggesting a possible additive effect of alcohol-related neurotoxicity and physiological aging. In older age groups, differences between cases and controls appeared attenuated, which may reflect the dominant influence of age-related axonal loss [25,27]. The absence of uniform thinning across all age strata, including the finding of comparable or slightly higher average pRNFL thickness in the 51-60-year group, may reflect biological variability, survivor bias, or the influence of unmeasured confounders, and underscores the exploratory nature of age-stratified subgroup analyses.

From a clinical perspective, these findings support the potential utility of SD-OCT as a non-invasive modality for identifying subclinical retinal structural changes in patients with ALD. However, the findings should be interpreted cautiously, given the study design and inherent limitations.

Limitations

This study has several limitations that should be considered when interpreting the findings. First, the retrospective single-center design limits causal inference and may restrict generalizability to broader populations. Second, clinical and anthropometric parameters such as body mass index were not consistently available and therefore were not included in the analysis. Additionally, disease severity stratification based on the Child-Pugh classification was not analyzed due to limited distribution across severity categories, which may have obscured severity-dependent effects on pRNFL thickness. Third, quantitative measures of alcohol exposure, including duration and cumulative lifetime intake, were not uniformly documented owing to the retrospective nature of the study, precluding dose-response analysis. Although age and sex were frequency-matched between groups and major systemic confounders such as diabetes and hypertension were excluded, other socioeconomic variables such as education level and rural residence differed between groups and were not adjusted for in multivariable models. These factors may influence nutritional status or healthcare access and could potentially confound pRNFL measurements. In addition, other potential confounders including smoking status and nutritional deficiencies were not analyzed, and IOP was not included as a covariate in the statistical analysis. Furthermore, multiple quadrant-wise comparisons were performed without formal correction for multiplicity, which may increase the risk of type I error. Finally, the absence of longitudinal follow-up precludes assessment of progressive retinal changes over time.

Future directions

Future studies should include prospective, longitudinal designs to evaluate the progression of RNFL thinning in patients with ALD. Incorporating quantitative measures of alcohol consumption, nutritional status, and smoking history may help clarify contributing risk factors. Multicenter studies with larger sample sizes and correlation with neuroimaging findings could further elucidate the hepatic-neural axis and validate SD-OCT as a screening tool for early neuroretinal damage.

## Conclusions

This study demonstrates an association between ALD and reduced pRNFL thickness, with more pronounced changes observed among younger adults. Age-stratified analysis revealed variable patterns of thinning, with quadrant-wise differences that were not uniformly significant across all age groups. The observed involvement of nasal and temporal quadrants may suggest a non-glaucomatous pattern of subclinical neuroretinal involvement; however, these findings should be interpreted cautiously and regarded as exploratory. Further prospective, longitudinal, and multicenter studies incorporating quantitative measures of alcohol exposure, disease severity stratification, and adjustment for potential confounding factors are required to better elucidate the underlying mechanisms and clinical significance of retinal structural changes in ALD.
